# Reduction of False Positives in Structure-Based Virtual Screening When Receptor Plasticity Is Considered

**DOI:** 10.3390/molecules20035152

**Published:** 2015-03-19

**Authors:** Yaw Awuni, Yuguang Mu

**Affiliations:** School of Biological Sciences, Nanyang Technological University, 60 Nanyang Drive, Singapore 637551, Singapore; E-Mail: yaw002@e.ntu.edu.sg

**Keywords:** virtual screening, receptor plasticity, false positives, docking, GOLD

## Abstract

Structure-based virtual screening for selecting potential drug candidates is usually challenged by how numerous false positives in a molecule library are excluded when receptor plasticity is considered. In this study, based on the binding energy landscape theory, a hypothesis that a true inhibitor can bind to different conformations of the binding site favorably was put forth, and related strategies to defeat this challenge were devised; reducing false positives when receptor plasticity is considered. The receptor in the study is the influenza A nucleoprotein, whose oligomerization is a requirement for RNA binding. The structural flexibility of influenza A nucleoprotein was explored by molecular dynamics simulations. The resultant distinctive structures and the crystal structure were used as receptor models in docking exercises in which two binding sites, the tail-loop binding pocket and the RNA binding site, were targeted with the Otava PrimScreen1 diversity-molecule library using the GOLD software. The intersection ligands that were listed in the top-ranked molecules from all receptor models were selected. Such selection strategy successfully distinguished high-affinity and low-affinity control molecules added to the molecule library. This work provides an applicable approach for reducing false positives and selecting true ligands from molecule libraries.

## 1. Introduction

Though currently it is a daunting task for any docking algorithm to practically explore the entire phase space of a protein receptor, there is enough evidence in support of the need to consider receptor flexibility in structure-based virtual screening (SBVS) in search of potential drug candidates [1,2,3]. It has been reported that rigid-receptor docking contributes significantly to inaccurate ligand binding energy estimations and poor binding mode predictions [4]. Although several strategies for incorporating receptor flexibility into molecular docking have been proposed, docking onto multiple receptor conformations (MRCs) is considered as the best option that allows one to take advantage of the full flexibility of the receptor [1]. In the MRCs technique, ligands are docked to multiple crystal, NMR or computer generated structures of the receptor. In one approach, usually referred to as ensemble docking [2], the ligands are docked to superimposed multiple conformations of the receptor. The docked solutions are then optimized to find the best fit between a ligand and a conformation. In another approach, which has been adopted in this study, ligands are docked separately onto each conformation of the receptor [5,6].

The use of computational techniques to generate multiple structures for proteins with few crystal or NMR structures has become popular. Even though there are several computational methods such as simulated annealing, Monte Carlo simulation and low-frequency normal modes for generating multiple conformations of a protein for SBVS, molecular dynamics (MD) simulation is commonly used. The MD structures are normally extracted from a trajectory of the apo [7] or the holo [5] form of the receptor. A recent study showed that MD structures are as good as crystal or NMR structures for SBVS [8].

One problem associated with using MRCs in SBVS is that each distinct conformation of the receptor that is added increases the number of false positives to handle [9]. Thus, selection of true ligands or potential drug candidates from the numerous false positives in a molecule library becomes difficult when receptor plasticity is considered. The burden and cost of having to deal with false positives is more pronounced where large molecule libraries are screened for potential drugs or lead molecules. In most cases, ligands selected from docking do not have inhibitory effects in experiments [10]; that is to say, most docking results turnout to be false positives. In this study, we relied on the binding energy landscape theory [11] and brought out a hypothesis that a true inhibitor can bind to different conformations of the binding site favorably. We devised a ligand-selection strategy that could facilitate the selection of true ligands from molecule libraries, by minimizing the inclusion of false positives into the selection, when receptor flexibility is considered in SBVS.

**Figure 1:**
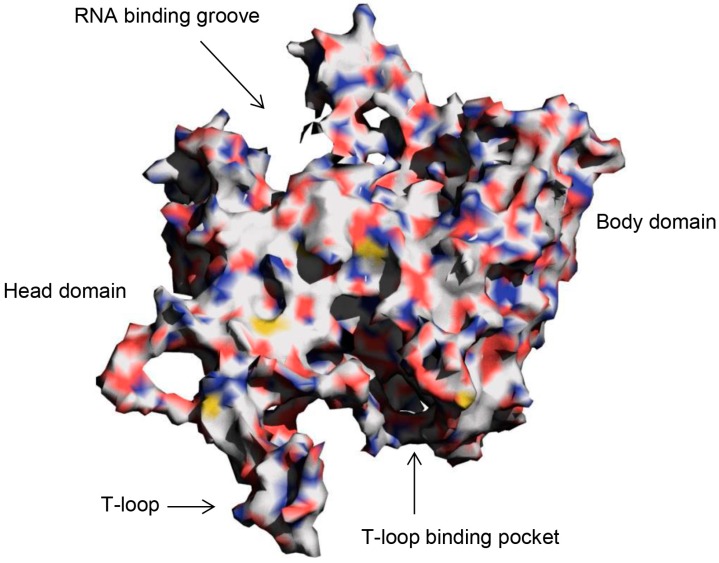
Surface representation of H1N1 Influenza A nucleoprotein (NP) monomer showing the head and body domains, T-loop, T-loop binding pocket and the RNA binding groove.

The influenza A nucleoprotein (NP), is a flu protein whose main role in the virus’ life cycle is to bind to the segmented RNA polymerase and its subunits (PA, PB1 and PB2) to form ribonucleoproteins (RNPs), which are then transported into the host cell’s nucleus where viral genome replication takes place. The protein is characterized by a head domain consisting of a tail-loop (T-loop) and a body domain within which are found the T-loop binding pocket and RNA binding groove [12] (see Figure 1). NPs, which are synthesized as monomers, aggregate into trimers before binding to RNA. The oligomerization process, a prerequisite for RNA binding, is aided by the insertion of the T-loop of one monomer into the T-loop binding pocket of a neighboring monomer [12]. Thus, small molecules that could inhibit NP oligomerization and/or RNA binding could be potential antiviral drug candidates. Studies have identified the T-loop binding pocket [12,13], RNA binding site [14] and a nucleozin binding site [15] of NP as targets for antiviral drug development. The fact that NP has different drug target sites partly informed our choice of it for this study.

## 2. Results and Discussion

Our ligand selection strategy sought to reduce the incidence of false positives in SBVS when receptor plasticity is considered. Relying on the binding energy landscape theory [11], the dynamic behavior of protein and successes chocked by attempts to account for receptor plasticity in the drug development process [2,3], we tested a hypothesis that a true binder fits favorably to varying conformations of the binding site. The hypothesis was tested by a search for potential small molecule inhibitors of influenza A NP oligomerization and RNA binding. We accounted for receptor plasticity by docking and scoring small molecules, including control molecules, in sets of six and five distinctive conformations of the T-loop binding pocket and the RNA binding site of NP respectively. To exclude false positives and select true ligands, the common members from all the top-ranked molecule lists obtained by docking the Otava PrimScreen1 diversity (OP1D) library, including control molecules, separately to all conformations of each binding site were selected. Thus, sets of six and five top-ranked lists were used for selecting molecules for the T-loop pocket and RNA binding site respectively. The selection strategy identified all control molecules that were added to the molecule library.

**Figure 2:**
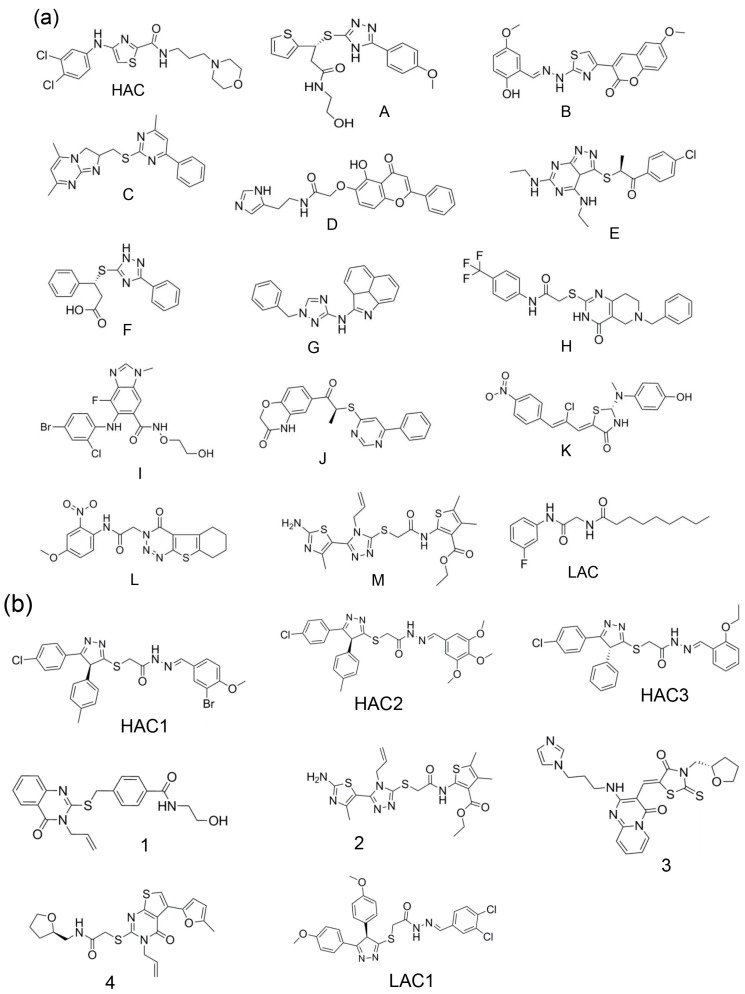
Molecules selected from the Otava PrimScreen1 library. (**a**) Selected molecules for the T-loop binding pocket; (**b**) Selected molecules for the RNA binding site. The LACS were not selected but are shown for illustrative purposes.

Table 1 shows the pattern of selection of molecules by screening the OP1D library with different conformations of the T-loop pocket and the RNA binding site of NP. As shown in Table 1a, only molecule **A** belongs to the top 50 molecules of all the six lists. The high-affinity control (HAC) met the selection criteria at the level of top 100 molecules. The selection of the HAC at top 100 but not top 50 molecules could be attributed to its reported low binding affinity [13] (see Table S1). Fourteen molecules were selected from the top 200 lists (see Figure 2a). It can also be seen in Table 1b that no molecule belongs to the top 10 molecules of all the five lists from the RNA binding site. The HACs (HAC1, HAC2 and HAC3) were selected at top 20, 30 and 50 respectively. Seven molecules were selected from the top 50 lists (see Figure 2b). The selection pattern of the control molecules is consistent with their experimentally determined binding affinities and/or activities [13,14] (See Table S1). The low-affinity controls (LACs) for both the T-loop binding pocket and RNA binding site were not selected because they were used to set the selection cutoff points. We believe that those molecules which were ranked as top solutions in some lists but ranked lower in other lists could be false positives.

**Table 1:** Pattern of selection of molecules from the Otava PrimSreen1 diversity library.

Selection for the T-Loop Binding Pocket		Selection for the RNA Binding Site	
Level of Comparison	Molecules Selected	Level of Comparison	Molecules Selected
Top-ranked 50	A	Top-ranked 10	-
Top-ranked 100	HAC and B	Top-ranked 20	HAC1
Top-ranked 150	C-E	Top-ranked 30	HAC2
Top-ranked 200	F-M	Top-ranked 40	1
-	-	Top-ranked 50	HAC3, 2-4
Total molecules selected	14	Total molecules selected	7

The emergence of few molecules in the intersection of top-ranked lists obtained by docking to different conformations of the T-loop binding pocket and the RNA binding site is consistent with reports that each distinctive conformation of the active site that is included into SBVS process brings on board its own false positives [9]. However, the emergence of the HACs as intersection molecules in the top-ranked lists give credence to our hypothesis that a true ligand fits favorably to different conformations of the binding site, and to our assertion that our selection criteria leads to the reduction of false positives encountered when receptor flexibility is considered in SBVS.

### 2.1. Selection by Docking to Multiple Receptor Conformations Narrows down Ligand Candidates

**Figure 3:**
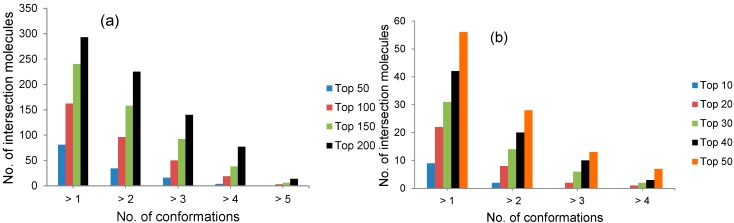
Number of intersection molecules in different number of conformations of the T-loop binding pocket and the RNA binding site of NP at different levels of comparison. (**a**) Number of intersection molecules for the T-loop binding pocket; (**b**) Number of intersection molecules for the RNA binding site.

To further show that our selection strategy identifies true ligands and reduces the false positives brought on board SBVS by distinct receptor conformations, we determined the number of intersection molecules in more than 1, 2, 3, 4 and 5 conformations of the T-loop pocket and RNA binding site where applicable (see Figure 3). As shown in Figure 3, the decrease in the number of intersection molecules as the number of conformations increase shows that the selection strategy can greatly narrow down the docking candidates. Since the fewer intersection molecules that emerged with increasing number of conformations do include the HACS (See Table 1), the observed trend could also mean that our selection criterion is a reasonable way to get rid of the false positives and select true ligands.

To get more accurate binding energies, the selected molecules in the top-ranked 100 molecules docked to the T-loop binding pockets and the entire selection for the RNA binding site were re-docked using the default setting (instead of the high throughput setting) of the GOLD genetic algorithm parameters [16]. The docking scores are listed in Table 2. As shown in Table 2, we found a more potent molecule, molecule **A**, ranking better than the HAC for binding to all the T-loop pockets, whereas for the RNA binding site, no molecule in the library was found to rank higher than the HACs in all conformations. In the following, we only focus on the results of the T-loop binding pockets.

**Table 2:** Default GOLD docking scores of selected molecules in all receptor conformations.

**Receptor Models for T-Loop Pocket**						
**Molecule**	**Crystal Structure**	**Structure 1**	**Structure 2**	**Structure 3**	**Structure 4**	**Structure 5**
A	74.17	92.21	75.70	78.95	78.85	66.77
B	65.98	70.28	82.14	78.00	74.54	66.03
HAC	63.24	76.84	64.20	77.06	78.08	64.59
**Receptor Models for RNA Binding Site**						
**Molecule**	**Crystal Structure**	**Structure 1**	**Structure 2**	**Structure 3**	**Structure 4**	
HAC1	70.08	67.94	64.90	69.46	64.19	
HAC2	64.63	64.70	64.08	65.64	65.77	
1	61.03	71.01	62.15	70.76	64.17	
HAC3	61.29	64.20	60.16	68.18	69.77	
2	59.38	60.57	60.64	61.42	65.58	
3	61.05	65.48	52.67	63.88	57.56	
4	60.00	59.41	56.82	60.59	64.08	

### 2.2. Molecule **A** Compared to the High-Affinity Control (HAC)

**Figure 4:**
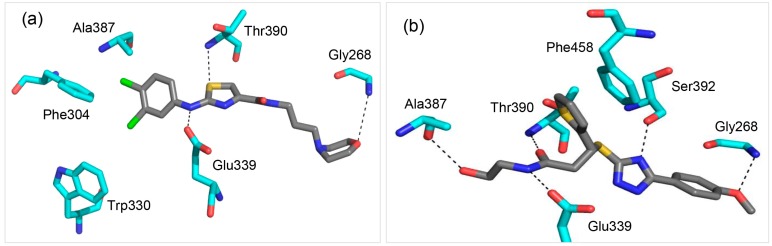
Predicted binding modes of the high-affinity control (HAC) and molecule **A** in the T-loop pocket of the crystal structure of NP. Carbon atoms of the two molecules are colored in dark grey while carbon atoms of NP residues interacting with the molecules are colored in cyan. Hydrogen bonds are represented by black dashes. (**a**) The HAC forms hydrogen bonds with Gly268, Glu339 and Thr390. Its dichloro-anilino group is involved in aromatic-π interactions with Phe304 and Trp330; (**b**) Molecule **A** forms hydrogen bonds with Glu339, Ala387, Thr390 and Ser392. Its thiophene group forms ring stacking with Phe458.

Previous work by Shen *et al.* [13] suggested that the NP inhibition mechanism by HAC could be emanating from its ability to interact with a Glu339 residue in the T-loop binding pocket. Therefore, to verify that the molecules selected by our criterion are not artifacts, we compared the binding modes and root mean square deviations (RMSDs) of the HAC and molecule **A** in the T-loop binding pocket of the crystal structure. Figure 4 shows the orientations of the two molecules as predicted by the GOLD docking program [17]. As shown in Figure 4a, the HAC forms hydrogen bonds with Gly268, Glu339 and Thr390. The dichloroanilino group is involved in aromatic-π interactions with Phe304 and Trp330. This predicted orientation is very close to that of Shen *et al*. [13]. The predicted binding mode of molecule **A** is shown in Figure 4b. The molecule forms hydrogen bonds with Glu339, Ala387, Thr390 and Ser392. The thiophene group of the molecule forms a ring stacking with Phe458. The ability of molecule **A** to form more hydrogen bonds could be one of the reasons why it obtains better GOLD scores than the HAC.

**Figure 5:**
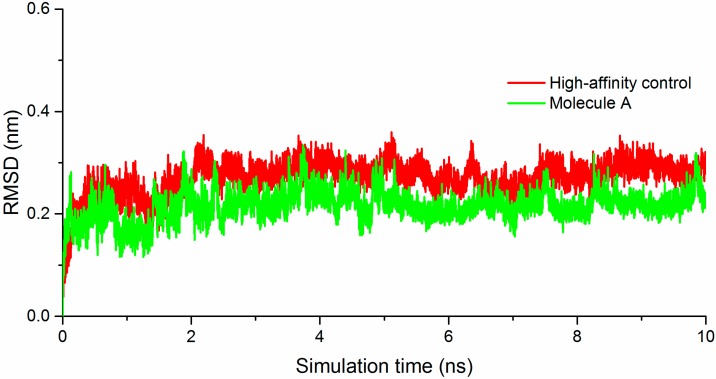
Molecular dynamics simulation data. RMSDs of the high-affinity control (HAC) and molecule **A** in complex with NP T-loop pocket are shown. The RMSDs were calculated on the molecules after least squared fitting of the protein.

To test the stability of the predicted binding modes of HAC and molecule **A**, we carried out a 10 ns simulation of each molecule in complex with the protein in explicit solvent. Figure 5 shows the RMSDs of the two molecules. They both bind stably with the protein with RMSD less than 0.35 nm. Molecule **A** has even smaller RMSDs. Hydrogen bond analysis showed that the high-affinity control makes three hydrogen bonds with the protein while molecule **A** makes four hydrogen bonds.

### 2.3. Glu339: A Hotspot for Molecule **A**

**Figure 6:**
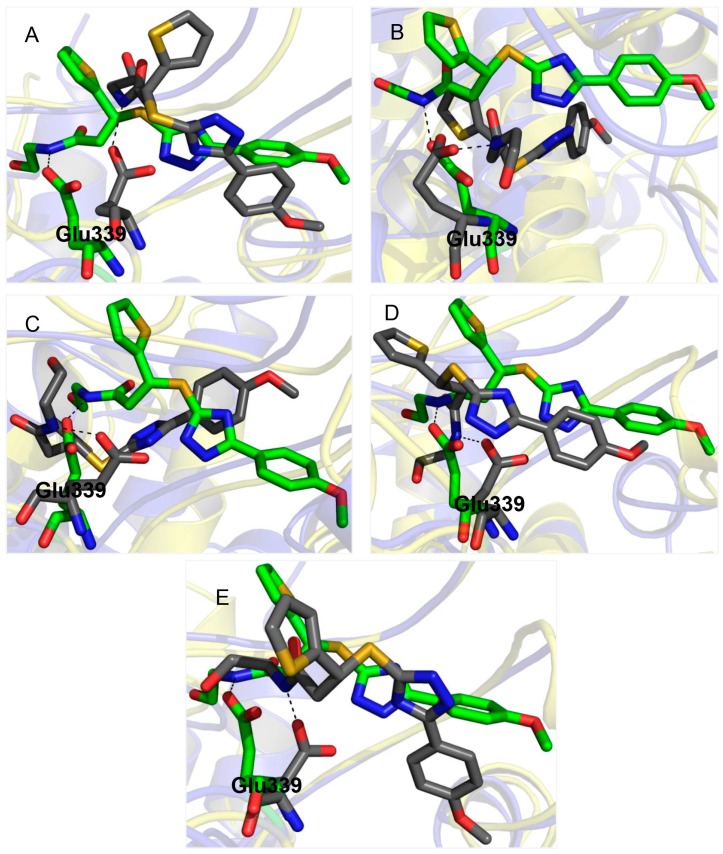
Binding modes of molecule **A** in the distinctive T-loop binding pocket conformations of NP. The ability of the molecule to interact with Glu339 in all T-loop binding pocket conformations is illustrated. The binding mode of the molecule with highest score in each conformation of the binding pocket was superimposed on that of the crystal structure. (**A**–**E**) represent structure 1, 2, 3, 4 and 5 superimposed on the crystal structure respectively. The crystal structure is colored in blue, MD generated structures are in yellow, carbon atoms of Glu339 and molecule **A** in the crystal structure are in green. Carbon atoms of Glu339 and molecule **A** in MD structures are colored in dark grey. Hydrogen bonds between Glu339 and molecule **A** are shown in black dashes.

By superimposing the protein-ligand complexes of the MD-generated T-loop-binding-pocket structures onto the crystal structure, the structural plasticity of the protein is illustrated (See Figure 6). In Figure 6, the predicted poses of molecule **A** are also shown. One can find that (i) Glu339 is one of the flexible residues in the NP T-loop binding pocket (ii) molecule **A** is able to make close contact with Glu339 in all conformations of the T-loop binding pocket through forming hydrogen bonds.

## 3. Experimental Section

### 3.1. Structure Preparation

The crystal structure of influenza A (H1N1) NP (PDB ID: 2IQH) was downloaded from the protein data bank. Since the structure is a homotrimer, a monomer consisting of chain A was generated by deleting chains B and C. The monomer was used for the study. All 73 missing residues were built in using the SWISS-MODEL package [18].

### 3.2. Molecular Dynamics Simulations

MD simulations were performed with Gromacs 4.5.4 [19], Amber 99SB force field [20], a time step of 0.002 ps and the Berendsen pressure coupler at a temperature of 300 K. The crystal structure was used as the seed structure for five independent 20 ns simulations. In each case, the protein was centered 10 Å from the edges of a cubic box and solvated in an explicit TIP3P water model [21]. The system was electrostatically neutralized by adding 16 chloride ions in 0.1 M NaCl. Energy minimization of the system was carried out using the steepest descent algorithm for 1000 steps with the maximum force threshold of 1.0 kJ/mol. The Particle Mesh Ewald algorithm was applied for the treatment of long range electrostatics with 10 Å for real-space cutoffs. The Van der Waals interactions were cut off at 14 Å. At 300 K, the system was equilibrated using NPT ensemble over 5 ns. Five independent MD trajectories of 20 ns each were generated with randomly assigned initial velocities. To adequately explore the phase space of the protein and to sample as many conformations as possible, the final configurations of the five simulations were taken as seed structures for producing another batch of five independent 20 ns trajectories with randomly chosen initial velocities. All 10 MD trajectories were then merged into a 200 ns combined trajectory.

### 3.3. Clustering for Distinctive Structures

The net 200 ns trajectory from the MD simulations contained 200,000 frames. Twelve selected amino acid residues within the T-loop binding pocket of NP were used as the reference group for structure superimposition and clustering for distinctive conformations of the pocket. These residues have been reported by an earlier study to be involved in the stabilization of NP-NP interactions [12]. They include Ser165, Arg267, His272, His334, Ser335, Glu339, Asp340, Ile347, Ile388, Arg389, Ser457 and Phe458. Using the gromos method of g_cluster utility in the Gromacs package [19] and a clustering RMSD cutoff of 0.25 nm, the 200 ns combined trajectory was grouped into five main clusters. The central structure of each of the five clusters was selected as the representative of the cluster. Also, 13 selected residues within the proposed binding site of HAC1 and its derivatives [14] were used as the reference group for structure superimposition and clustering for distinctive conformations of the RNA binding site of NP. These residues include Arg174, Arg175, Ser176, Gly177, Ala178, Ala179, Gly180, Ala181, Ala182, Val183, Lys184, Met189 and Glu192. Using a clustering RMSD cutoff of 0.18 nm, the 200 ns combined trajectory was grouped into four main clusters. The central structure of each of the four clusters was selected as the representative of the cluster. Thus, sets of five and four distinctive MD structures of the T-loop pocket and the RNA binding site, respectively, were selected for further study.

### 3.4. Docking and in Silico Screening

All dockings were performed with the GOLD program [17]. Flexible ligands were docked onto rigid but distinct conformations of each receptor. Sets of six and five distinctive structures of the T-loop binding pocket and RNA binding site (the MD generated structures plus the crystal structure), respectively, were separately used as receptor models in the docking process. The receptors were prepared with the automatic protein preparation tool of Accelrys Discovery studio^TM^ 3.5(DS) [22], in which protons and partial charges were added. All prepared proteins were saved as mol2 files. The OP1D library was prepared for docking with the automatic ligand preparation tool of DS [22]. Protonation of the molecules was done within a pH range of 6.5 to 8.5. In total, 1000 molecules were prepared for the OP1D library. Two molecules whose binding affinities for NP T-loop pocket have been measured in previous experiments [13] were added to the library to serve as the HAC and LAC for the T-loop pocket (See Table S1). Similarly, four molecules, all derivatives of 3-mercapto-1,2,4-triazole, that have been shown to inhibit the influenza virus by binding to the RNA binding site of NP [14] were added to serve as controls. Three of the four molecules served as HACs (HAC1, HAC2 and HAC3) while one served as the LAC for the RNA binding site (See Table S1). Partial charges of the ligand atoms were computed using Antechamber utility [23] in AMBER10 package [24]. The NP T-loop binding pocket was defined as a sphere of radius of 10 Å centered on the His272 amino acid residue. The RNA binding site, on the other hand, was defined as a sphere of radius of 10 Å centered on Arg174 amino acid residue. The GOLD genetic algorithm parameters were set to the virtual screening setting [16]. The prepared molecule library was docked separately to each distinctive receptor conformation of the T-loop binding pocket and the RNA binding site.

### 3.5. Ligand Selection Criteria

Selection of molecules from the docking results was guided by the following strategy. Ligands were sorted in decreasing order of GOLD scores in docking exercises targeted at MRCs of the T-loop binding pocket and the RNA binding site of NP as described earlier. Sets of six and five sorted ligand lists for the T-loop pocket and RNA binding site were created, respectively. With regards to the T-loop binding pocket, the common molecules that appeared in the six sorted lists as the top-ranked 50 molecules, 100 molecules, 150 molecules, *etc.*, were selected. Selection was stopped at the top *n* 50 when the LAC emerged at the top (*n* + 1) 50. For the RNA binding site, the common molecules that appeared in the five sorted lists as top-ranked 10 molecules, 20 molecules, 30 molecules *etc.*, were selected. Selection was stopped at the top *n* 10 when LAC1 emerged at the top (*n* + 1) 10. Thus, we set the cutoff as the common top 200 and top 50 molecules for solutions from the T-loop binding pocket and the RNA binding site, respectively. 

### 3.6. Protein-Ligand Complex Simulations

The basic MD protocol was the same as described in the previous section. The protein topology was built with pdb2gmx utility in Gromacs [19] using the Amber 99SB force field [20] and TIP3P water model [21] for solvation. Quantum mechanical calculations for partial charges of ligands were performed with Gaussian09 [25], using the R.E.D.-III.4 tool [26]. Atom types were assigned by the antechamber utility [23] included in the AMBER10 package [24]. The topologies of ligands were built for the general amber force field (GAFF). Simulations of 10 ns each were performed for selected protein-ligand complexes.

## 4. Conclusions

Our goal was to devise strategies to reduce the false positives encountered when receptor plasticity is incorporated into SBVS. Distinctive multiple conformations of the T-loop binding pocket and RNA binding site of NP were used as receptor models in docking exercises for scoring molecules in the OP1D library including control molecules. Based on the docking scores, sets of six and five separate sorted-molecule lists were created for the T-loop pocket and the RNA binding site, respectively. The common molecules that sat in the top-ranked solutions of each set of lists were selected.

Our selection criteria identified all control molecules added to the library in a manner that is consistent with their experimentally determined binding affinities and/or activities. Molecule **A**, one of the selected molecules for the T-loop binding pocket, earned better GOLD docking scores than the HAC in all conformations of the pocket. The following MD simulations showed that the binding mode predicted by the GOLD docking program can be maintained stably during 10 ns trajectory. Molecule **A** could also interact with Glu339 in all conformations of the binding pocket. This behavior of the molecule satisfies a requirement suggested by Shen *et al.* [13] that molecules that interact with Glu339 could inhibit NP oligomerization.

The screening and selection criteria described here provide a means for selecting true ligands from a molecule library by reducing the false positives that are usually encountered when receptor plasticity is considered in SBVS. The notion that a true binder fits favorably to different conformations of the binding site roots in the energy landscape theory [27]. Binding, like protein folding, has a rugged funnel-like free energy landscape to allow multiple interaction patterns [11]. Structural plasticity of proteins is a natural phenomenon. Theoretically, however, it induces perturbations to the rigid docking process. Our criterion selects molecules which are immune to the protein structural variations. In such a way, our method reconstructs the binding energy landscape which is a necessity to describe a true binding process.

## Supplementary Materials

Supplementary materials can be accessed at: http://www.mdpi.com/1420-3049/20/03/5152/s1.
