# Supplementary material for: Reduction of False Positives in Structure-Based Virtual Screening When Receptor Plasticity Is Considered
Source: Molecules. 2015 Mar 19;20(3):5152–64. doi: 10.3390/molecules20035152 (PMC6272817; doi:10.3390/molecules20035152)
Supplement: Supplementary file 1 [file molecules-20-05152-s001.pdf]

Supplementary Materials

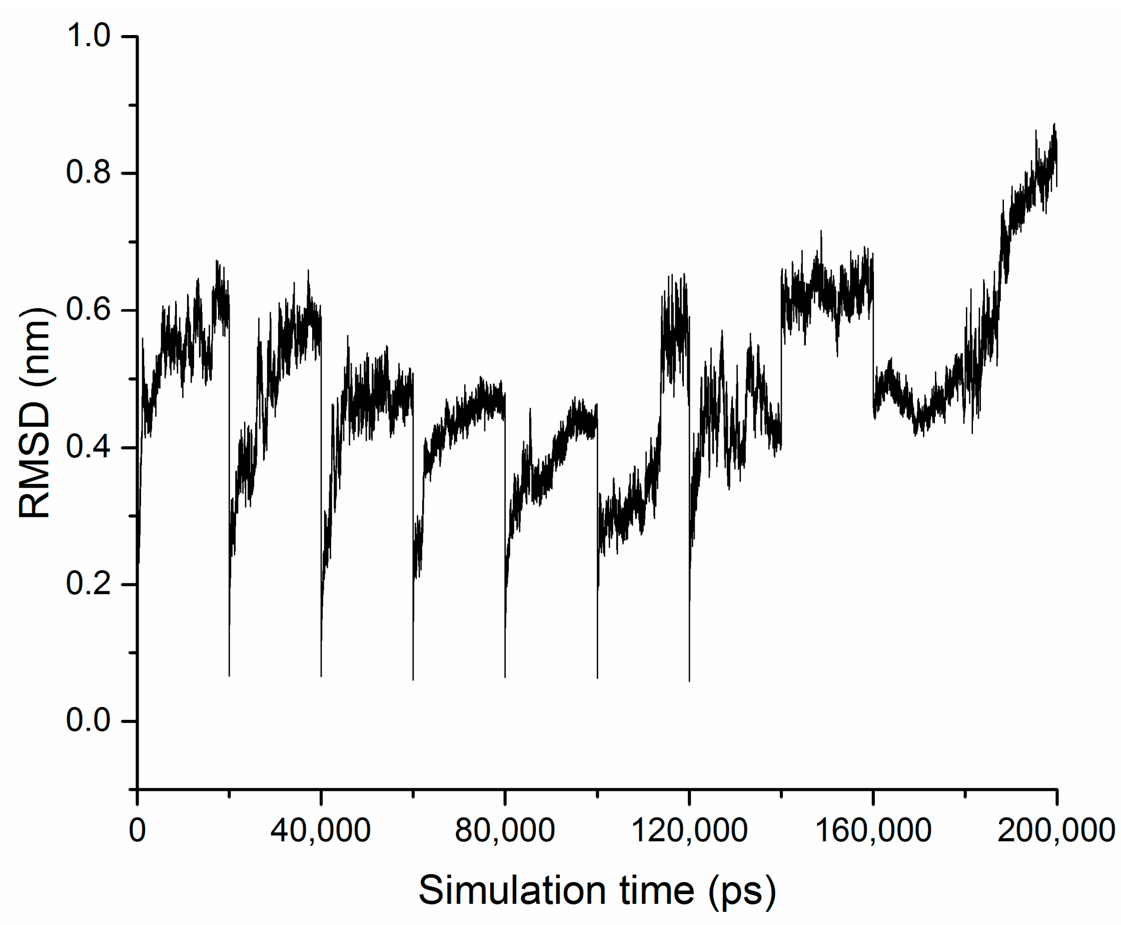

Figure S1. RMSD of NP backbone in the 200,000 frames used for clustering. The first 100,000 frames show the RMSD of the first five independent simulations of the crystal structure. The last 100,000 frames show the RMSD of independent simulations of the final structures of the first five simulations.

Table S1. Control molecules and their experimentally determined affinities/activities [13,14]. The T-loop binding pocket and RNA binding site are conserved in type A influenza stains, including H1N1 and H3N2.

**Control Molecules for the T-Loop Binding Pocket**		
Control	Target (H1N1)	Activity (IC_{50})/µm
HAC	T-loop binding pocket	2.70
LAC	T-loop binding pocket	37.50
**Control Molecules for the RNA Binding Site**		
Control	Target (H3N2)	Activity (# of Plaques/Oseltamivir)
HAC1	RNA binding site	0%
HAC2	RNA binding site	4%
HAC3	RNA binding site	4%
LAC1	RNA binding site	5%

Table S2. Number of intersection molecules in different number of conformations of the T-loop binding pocket and RNA binding site of NP at different levels of comparison.

**Intersection Molecules for the T-Loop Binding Pocket**					
	No. of Conformations				
Level	>1	>2	>3	>4	>5
Top 50	81	34	16	4	1
Top 100	162	96	50	19	3
Top 150	240	158	92	38	6
Top 200	293	225	140	77	14
**Intersection Molecules for the RNA Binding Site**					
	No. of Conformations				
Level	>1	>2	>3	>4	
Top 10	10	2	0	0	
Top 20	22	8	2	1	
Top 30	31	14	6	2	
Top 40	42	20	10	3	
Top 50	56	28	13	7	
